# Effect of Chitosan and Amphiphilic Polymers on the Photosensitizing and Spectral Properties of Rose Bengal

**DOI:** 10.3390/molecules27206796

**Published:** 2022-10-11

**Authors:** Anastasia S. Kuryanova, Marina A. Savko, Vladislav S. Kaplin, Nadezhda A. Aksenova, Victoria A. Timofeeva, Aleksandr V. Chernyak, Nicolay N. Glagolev, Petr S. Timashev, Anna B. Solovieva

**Affiliations:** 1 N.N. Semenov Federal Research Center for Chemical Physics, Russian Academy of Sciences, Kosygina St. 4, 119991 Moscow, Russia; 2 Institute for Regenerative Medicine, Sechenov University, Trubetskaya St. 8-2, 119991 Moscow, Russia; 3 Federal Research Center of Problem of Chemical Physics and Medicinal Chemistry, Russian Academy of Sciences, Ac. Semenov Avenue 1, 142432 Chernogolovka, Russia; 4 World-Class Research Center “Digital Biodesign and Personalized Healthcare”, Sechenov First Moscow State Medical University, Trubetskaya St. 8-2, 119991 Moscow, Russia; 5 Chemistry Department, Lomonosov Moscow State University, Leninskiye Gory 1-3, 119991 Moscow, Russia

**Keywords:** rose bengal, chitosan, amphiphilic polymers, tryptophan, fluorescence anisotropy, NMR, lactone form of the dye

## Abstract

The influence of chitosan (CS) and amphiphilic polymers (AP: pluronic F108 and polyvinylpyrrolidone (PVP)) on the photocatalytic activity of rose bengal (RB) in a model reaction of tryptophan photo-oxidation in phosphate-buffered saline (PBS) was studied. It was shown that in the presence of CS, the effective rate constant ***k****_eff_* of tryptophan photo-oxidation catalyzed by RB in PBS solution decreases by a factor of two. This is due to the ionic interaction of the RB with the chitosan. Rose bengal in a slightly acidic environment (pH 4.5) passes into a neutral lactone form, which sharply reduces the photosensitizing properties of the dye. It was demonstrated that the introduction of AP into a solution containing RB and CS prevents direct interaction between RB and CS. This is evidenced by the presence of photocatalytic activity of the dye in the RB-AP-CS systems, as well as bathochromic shifts of the main absorption bands of the dye, and an increase in the optical density and luminescence intensity of the RB when AP is introduced into a buffer solution containing RB and chitosan. The presence of RB-CS and RB-AP interaction in aqueous and PBS media is confirmed by the increase in the degree of fluorescence anisotropy (***r***) of these binary systems. In an aqueous solution, the value of ***r*** for the RB-F108-CS system decreases by a factor of 3.5 (compared to the value of *r* for the RB-CS system), which is associated with the localization of the dye in pluronic micelles. In PBS, the fluorescence anisotropy is practically the same for all systems, which is related to the stability of the dye structure in this medium. The presence of interaction between RB and AP in aqueous solutions was confirmed by the proton NMR method. In addition, the formation of RB-F108 macromolecular complexes, which form associates during solution concentration (in particular, during evaporation), was shown by AFM. Such RB-AP-CS systems may be promising for practical application in the treatment of local foci of infections by aPDT.

## 1. Introduction

Photodynamic therapy (PDT) is one of the safest, most minimally invasive methods for the treatment of localized oncological tumors, hard-to-heal wounds, trophic ulcers, and a number of autoimmune diseases [1,2,3,4]. The mechanism of photodynamic action is quite complex and not fully understood. Molecules of an excited sensitizer can directly interact with the substrate (biomolecules) with the transfer (detachment) of electrons and the formation of intermediate radical products, which then interact with oxygen, generating the formation of reactive oxygen species (ROS: ^1^*O*_2_, *O*_2_*^−^*, *OH^−^*), (type *I* photochemical reaction) [2,5]. An alternative way is direct triplet–triplet energy transfer between molecules of an excited photosensitizer (^3^PS*) and molecular oxygen, which leads to the generation of singlet oxygen ^1^*O*_2_ (type *II* photochemical reaction) [6].

One of the directions of PDT, which is currently being intensively developed, is photodynamic inactivation of pathogenic microorganisms or antimicrobial PDT (aPDT), since in vitro and in vivo studies demonstrated that bacteria, viruses and fungi (including those with multiple antibiotic resistance) are capable of accumulating PS and undergoing photodynamic degradation [7,8,9].

The most effective and non-toxic PS are porphyrins, chlorins and their derivatives (foscan, lutex, tookad, photofrin), which are currently used in the clinical practice of PDT [2,10,11]. Recently, along with porphyrin photosensitizers, a number of dyes of synthetic and natural origin (methylene blue, toluidine blue, rose bengal, curcumin, hypericin) capable of generating ROS upon excitation have been used in aPDT practice [12,13,14,15,16]. Such dyes are more accessible than porphyrins and practically do not concede to them in activity. It is important to note that many dyes have their own bactericidal activity, which can increase the effectiveness of PDT in the treatment of localized infections [16,17].

One of the problems of the application of sensitizers in photodynamic therapy, which has not been solved so far, is the insufficient effectiveness of PS in the processes of in vivo destruction of pathological cells and tissues. This problem is due to the high hydrophobicity of the structures of porphyrin and non-porphyrin PS, causing their aggregation in aqueous media (even at relatively low concentrations in solution (≥1 × 10^−6^ M)) [18]. To prevent this phenomenon, PS are obtained in the form of covalently (or non-covalently) bound conjugates with biologically active molecules (natural or synthetic), which, on the one hand, prevent PS aggregation and, on the other hand, facilitate the penetration of PS into the cell. Usually, PS conjugates are obtained with proteins, lipids, and even fullerenes [19,20,21,22].

In addition, to increase the effectiveness of the PDT method, other methods of influencing pathological tissue areas, in particular, thermotherapy or exposure to biologically active compounds (enzymes, antioxidants, wound healing substances) are simultaneously used [21,23,24].

Previously, we showed that some amphiphilic polymers (AP) (pluronics, polyvinylpyrrolidone, polyethylene glycol) are able to increase the photodynamic activity of PS by forming weakly bound complexes with photosensitizers in aqueous solutions (as shown by the ^1^H NMR method) and thereby initiating their disaggregation [25]. The experiments were carried out under model conditions (in the tryptophan photo-oxidation reaction), in vitro—on cell cultures (fibroblasts, adenocarcinoma of the human breast and liver), and in vivo—in the treatment of infected wounds in Wistar rats [26,27,28]. In addition, during PDT treatment (using the photoditazin–pluronic F127 complex as a PS) of a full-thickness excision wound model in rats, chitosan polysaccharide was added to the photoditazin–pluronic F127 complex to increase the effectiveness of the therapy, which led to a significant improvement in the condition of the wound surface [28].

In this work, we studied the effect of chitosan (CS) on the photosensitizing activity of rose bengal (RB) in generation of singlet ^1^*O*_2_ oxygen in the reaction of tryptophan photo-oxidation, in the presence and absence of amphiphilic polymers in phosphate-buffered saline (PBS). Possible interactions of polymer components of the system with the dye were also analyzed by optical and fluorescence spectroscopy in order to reveal the possibility of such interactions affecting the photodynamic activity of RB. Recently, rose bengal has been one of the most studied dyes with photosensitizing properties for practical use in aPDT [17,29,30]. The main advantages of RB are the high quantum yield generation of singlet oxygen ^1^*O*_2_ (Δ = 0.76), its own bactericidal activity, and low cost of this dye [31].

Rose bengal is a dianion dye with a fluoronic structure, prone to the formation of strong aggregates with low PS activity. In addition, due to its electronegativity, RB practically does not penetrate the bacterial cell membrane, the surface of which is known to be negatively charged. To improve the penetration of RB into the cell, it can be used in combination with a cationic polymer [32]. In this work, chitosan was chosen as a cationic polymer. It should also be noted that RB has long been used in medical practice in the diagnosis and treatment of eye pathologies, and is also an effective PS drug in the treatment of diseases of the oral cavity [33,34,35].

## 2. Results and Discussion

### 2.1. Effect of Polymers on the Photocatalytic Activity of RB

**Figure 1:**
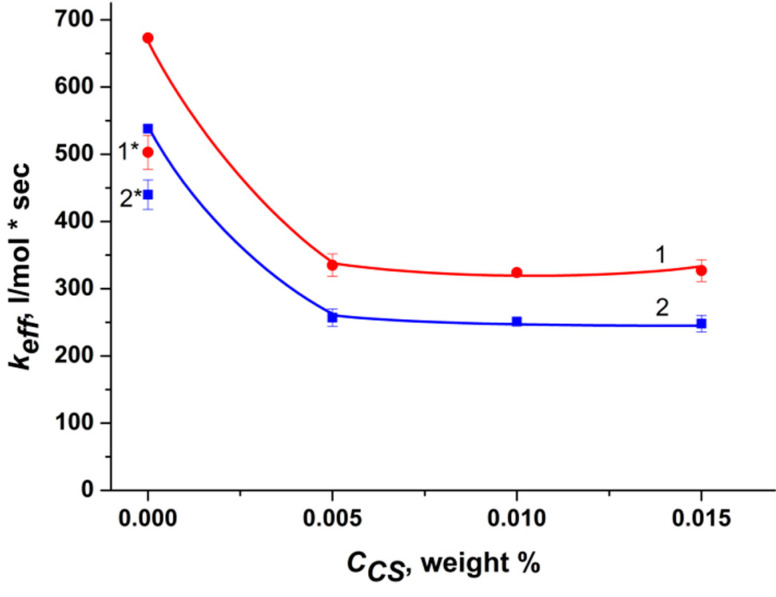
Dependence of ***k****_eff_* of Trp photo-oxidation in the presence of RB in PBS medium on the chitosan concentration. Points 1* and 2* are the rate constants (***k****_eff_*) of photo-oxidation of Trp catalyzed by RB in PBS medium in the presence of 0.1% acetic acid. [RB] = 2 × 10^−6^ M (1 and 1*) and 5 × 10^−6^ M (2 and 2*), [Trp] = 1 × 10^−4^ M.

Figure 1 shows the dependence of the effective rate constant ***k****_eff_* of the tryptophan photo-oxidation reaction in the presence of RB (2 and 5 × 10^−6^ M) on the concentration of chitosan (curves 1 and 2, respectively).

It can be seen that even at the minimum concentration of chitosan in the solution (5 × 10^−3^ wt.%), the ***k****_eff_* value decreases by about a factor of two (in proportion to the initial RB concentration), but a further increase in its content no longer affects the rate constant. Therefore, further kinetic and spectral studies were carried out at a given chitosan concentration of 5 × 10^−3^ wt.%.

**Scheme 1:**
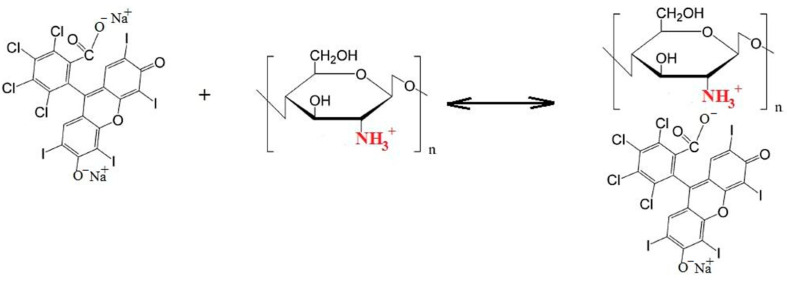
Ionic interaction between RB and CS.

Obviously, the observed dependencies indicate the interaction of RB dianions with protonated amino groups of chitosan and the existence of the following equilibrium (Scheme 1) [36]:

The cationic nature of chitosan is quite specific, since most polysaccharides are usually either neutral or negatively charged in an acidic environment. The interaction of chitosan with anionic compounds (synthetic and natural polymers, dyes) can lead to the formation of electrostatic complexes. The resulting composites based on polyelectrolytes are promising for practical applications in medicine [32,37,38].

Thus, RB molecules that are not bound to the chitosan polymer chain retain their photocatalytic activity. It is obvious that this equilibrium can exist only in a buffer medium, where the pH for the RB–CS system is 4.5. Indeed, it turns out that when this reaction is carried out in water, the introduction of chitosan leads to almost complete binding of RB by the chitosan polymer chain and a drop in the photocatalytic activity of RB by a factor of 25 (almost to zero). Since chitosan dissolves only in a slightly acidic medium (pH = 4.5), the effect of acetic acid in PBS medium on the ***k****_eff_* value at RB concentrations of 2 × 10^−6^ M and 5 × 10^−6^ M at pH = 4.5 (Figure 1, points 1* and 2 *) was studied. It can be seen that in a slightly acidic medium, a decrease in the value of ***k****_eff_* or photocatalytic activity of RB by approximately 1.3 times is also observed. As mentioned above, this is associated with the formation of an inactive lactone form of the dye [39,40].

**Figure 2:**
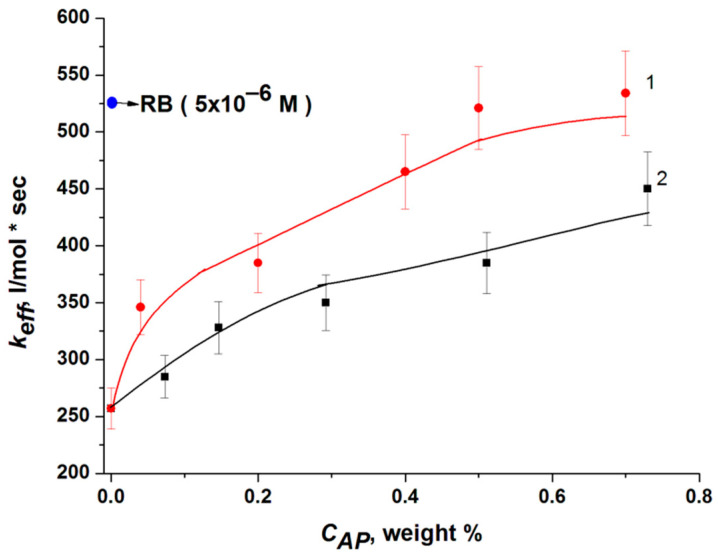
Dependence ***k****_eff_* of Trp photo-oxidation (1 × 10^−4^ M) in the presence of RB (5 × 10^−6^ M) and chitosan (0.005 wt.%) with the addition of PVP (1) and pluronic F108 (2) on the concentration of polymers: [PVP] = 0–0.7 wt.% and [F108] = 0–0.73 wt.%. The dye and AP were dissolved in PBS medium.

However, it was shown that RB binding to chitosan macromolecules can be prevented by introducing amphiphilic polymers (AP) into the system. As follows from Figure 2, pluronic F108 and PVP almost completely block the interaction of RB with CS and restore the photocatalytic activity of the dye.

### 2.2. Effect of the Nature of Polymers on the RB Absorption and Fluorescence Spectra

It should be noted that the interaction of RB with chitosan and AP is reflected in the electronic absorption spectra (EAS) and dye fluorescence. Since the interaction of RB with polycationic CS leads to the formation of a photoinactive form of the dye, the optical density of RB absorption bands in the buffer solution and in water decreases in the presence of chitosan (Table 1). As follows from Table 1, a similar drop in the optical density of the RB absorption bands is also observed in a weakly acidic medium.

There are two bands in the absorption spectrum of RB: the main one with a maximum at λ = 550 nm and a “shoulder” in the region of λ = 515 nm, which are attributed to the monomeric and dimeric forms of the dye, respectively [41,42]. The formation of stable dimeric (or more complex) forms in aqueous solutions is one of the characteristic features of fluoronic dyes, including rose bengal. This is due to the high hydrophobicity of the structure of dyes, which are based on dibenzopyran (xanthene) [18].

The presence of chitosan or acetic acid and the formation of the lactone form of the dye also leads to a decrease in the RB luminescence intensity by 1.5–2 times (Table 2). At the same time, it is natural that the indicated decrease in the optical density of absorption bands and luminescence intensity in the presence of chitosan and acetic acid is most pronounced in aqueous solutions compared to buffer solutions (Table 1 and Table 2).

**Figure 3:**
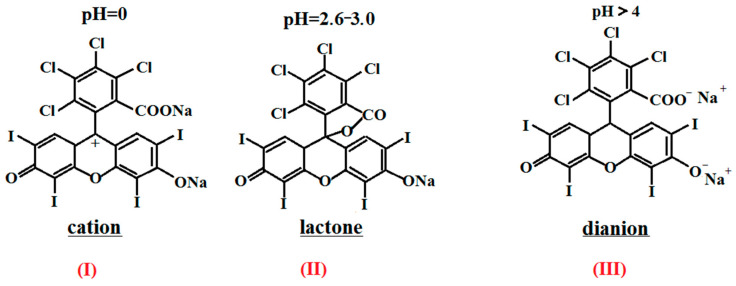
Tautomeric forms of rose bengal.

It is known that, depending on the pH of the medium, RB can exist in cationic, neutral (lactone), and anionic forms (Figure 3). [36,37]. In a neutral photoinactive form, rose bengal exists in a slightly acidic environment (pH < 5) (Figure 3, II). In a neutral and slightly alkaline medium (pH 7.2–7.6), RB has a characteristic absorption spectrum with two bands (550 and 515 nm), corresponding to the dianion form of rose bengal (Figure 3, III). Thus, RB works as a PS only in a neutral and slightly alkaline environment (pH 7.2–7.6).

Thus, in the presence of chitosan, a decrease in the optical density of the absorption bands and a drop in the intensity of the fluorescence bands are observed. At the same time, the position of the absorption and fluorescence bands in the corresponding spectra does not change, which indicates the absence of intermolecular interactions of the fragments of dye molecule, i.e., the fluorophore with the functional groups of polysaccharide [26].

However, the introduction of amphiphilic polymers into solutions containing RB and chitosan causes bathochromic shifts of the absorption bands in the EAS and fluorescence spectra of the system. Previously, we demonstrated that in the presence of AP-PVP and pluronic F108, bathochromic shifts of both RB absorption bands by 5–15 nm and an increase in the optical density and intensity of the fluorescence band are observed in the EAS of RB, which was interpreted as the process of dye disaggregation in the presence of AP [43].

**Figure 4:**
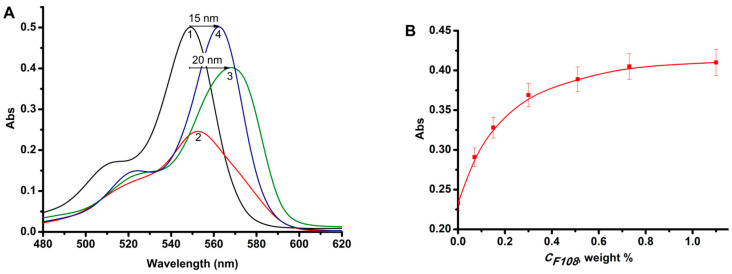
(**A**) Electronic absorption spectra of RB C = 5 × 10^−6^ M in PBS (1), RB-CS (2), RB-F108-CS (3), RB-PVP-CS (4), where [F108] = 0.73%, [PVP] = 0.2% and [CS] = 0.005%. (**B**) Dependence of the optical density of electronic absorption spectrum of the RB-CS system with the addition of F108 on the concentration of pluronic (0–1.1 wt.%).

**Figure 5:**
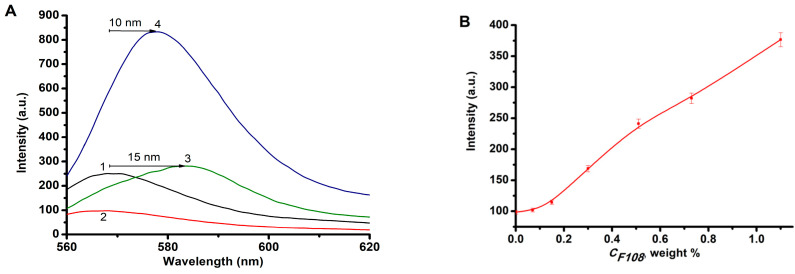
(**A**) Fluorescence spectra of RB C = 5 × 10^−6^ M in PBS (1), RB-CS (2), RB-F108-CS (3), RB–PVP-CS(4), where [F108] = 0.73%, [ PVP] = 0.2% and [CS] = 0.005%. (**B**) Dependence of the fluorescence intensity of the RB-CS system with the addition of F108 on the pluronic concentration (0–1.1 wt.%).

In this case, when amphiphilic polymers (pluronic F108 and PVP) are added to a solution in PBS containing RB and CS (Figure 4A), a bathochromic shift by 20 nm of the absorption band with λ = 550 nm (compared to the RB band in PBS in the absence of AP and CS, curve 1) in the presence of pluronic F108 (and by 15 nm in the presence of PVP), as well as an increase in optical density, are observed. In addition, there is an increase in intensity and a bathochromic shift of the RB fluorescence bands (by 10–15 nm) in the presence of chitosan and both APs (Figure 5A). At the same time, an increase in the content of pluronic F108 (Figure 4B) does not allow it to reach the initial values of the optical density of pure RB. Perhaps this is due to the ionic binding of the dye with chitosan and partial destruction of the original micellar structure of pluronic in a slightly acidic environment (pH = 4.5) [44,45]. The optical density values for RB and RB-PVP-CS practically coincide (Figure 4A, curves 1 and 4). Most likely, rose bengal forms sufficiently strong hydrophobic bonds with PVP, which partially prevents the formation of the RB-CS complex [46].

To reveal the features of intermolecular interactions of rose bengal with amphiphilic polymers and polysaccharides in aqueous/buffer solutions, the effect of PVP, pluronic F108, and chitosan on the degree of RB fluorescence anisotropy were studied (Table 3). According to the table, the fluorescence anisotropy (***r***) of pure rose bengal in aqueous and PBS solution is relatively small (0.26–0.27), which is consistent with data in the literature [47], and the addition of polymers has a different effect on the value of ***r*** in water and in a buffer solution. As can be seen, the value of ***r*** in the buffer solution practically does not change in the presence of polymers, which obviously indicates a greater stability of the fluorophore structure in a phosphate salt solution with I = 0.14 mol/L [48] and, consequently, the lower sensitivity of the anisotropy ***r*** to the molecular environment compared to the corresponding aqueous solutions. Indeed, in an aqueous solution in the presence of chitosan, due to ionic interactions with protonated amino groups of the polysaccharide, the freedom of rotation of the fluorophore decreases, which leads to an increase in the fluorescence anisotropy [49]. The value of ***r*** practically does not change in the presence of acetic acid. An increase in the anisotropy of RB fluorescence is also observed in the presence of AP, primarily in solutions with PVP, which indicates the presence of a noticeable interaction of the dye with polyvinylpyrrolidone. In this case, the dye and PVP have noticeably stronger interactions than RB with pluronic and, accordingly, the growth of ***r*** in the presence of PVP is significantly greater than in the RB-F108 system, which also corresponds to the above spectral data.

Interestingly, when amphiphilic polymers F108 and PVP are added to an aqueous solution of RB and chitosan with ***r*** = 0.32, the anisotropy drops by 3.5 and 1.3 times, respectively. Obviously, AP interacting with the dye provides it with greater mobility due to the breaking of ionic bonds with chitosan. This is especially evident in the case of the micelle-forming pluronic F108, where RB is in the free molecular state in the volume of micelles. A 1.2-fold decrease in the RB fluorescence anisotropy in the RB-F108-CS system is observed even in a buffer solution. It is important to note that the decrease in fluorescence anisotropy in the presence of AP occurs mainly for micellar-forming F108; for PVP, this effect is not so pronounced. This again emphasizes that the degree of fluorescence anisotropy decreases for non-aggregated fluorophore molecules in their dilute solutions.

**Figure 6:**
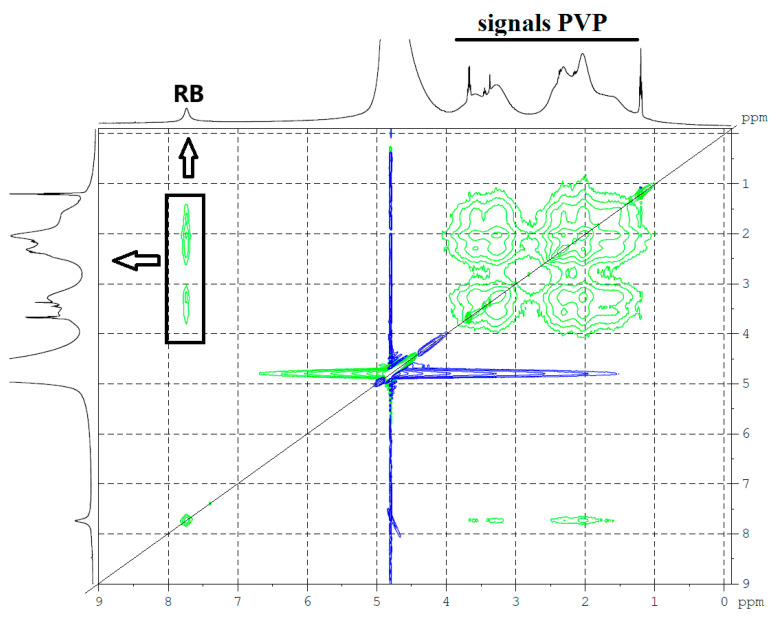
^1^H-^1^H NEOSY spectrum of the RB-PVP solution of 1:1 ratio in deuterated water.

The presence of interaction between RB and PVP is also revealed in the study of the NMR spectra of these systems. Figure 6 demonstrates two-dimensional ^1^H NOESY (Nuclear Overhauser Exchange Spectroscopy) spectrum of a deuterated aqueous solution of RB and PVP in a mass concentration ratio of 1:1. The proton spectra of this two-component system are plotted along the axes. The two-dimensional spectrum is symmetrical with respect to the diagonal (thin black line in the spectrum). The diagonal line is the proton spectrum. Off-diagonal correlation signals in the spectrum are the result of the Overhauser effect and/or chemical exchange. Chemical exchange between the aromatic protons of RB (about 7.7 ppm) and the protons of the PVP polymer is unlikely. Therefore, the signals in the highlighted area in Figure 6 (Overhauser effect) carry information about the intermolecular interaction (overlapping of electron clouds) of RB and PVP protons, which is possible due to their spatial proximity. Most likely, stack structures are formed. In addition to broadening in the presence of BR, the ^1^H spectra of PVP signals exhibit a slight shift towards strong fields, which is typical for the case when PVP protons are partially screened by the system of aromatic rings of RB molecules in the stack.

**Figure 7:**
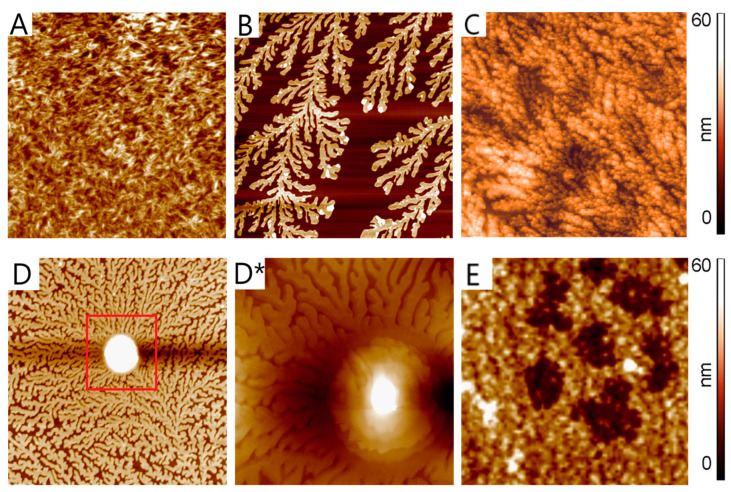
AFM images of the surface areas of the films in topography mode: (**A**) RB, (**B**) F108, (**C**) CS, (**D**,D*) RB–F108 (1:10) and (**E**) RB–F108–CS (1:10:2). The image size was of 10 × 10 μm for (**A**–**E**) and 3 × 3 μm for (D*).

The AFM method was used to study the surface of thin films obtained on mica from solutions of RB, pluronic F108 and CS, as well as from solutions of their double (RB-F108 = 1:10) and ternary (RB-F108-CS = 1:10:2) mixtures. The surface structure of RB films is represented by elongated cylindrical formations (Figure 7A). Pluronic F108 and chitosan form a dendrite-like structure on the mica surface (Figure 7B,C). At the same time, in comparison to pluronics, CS demonstrates a denser packing of dendrites with a fine-grained structure, which is probably due to the high MM of chitosan (50–190 kDa) compared to pluronic F108 (MM 14.6 kDa) [50,51]. In the presence of RB, significant changes in the surface structure of the polymer are observed (Figure 7D,D*). Convex formations of a spheroid shape appeared on the surface of pluronic F108. The layered structure of the new formation is visible, which possibly reflects the formation of RB-F108 agglomerates. When chitosan is introduced into the two-component RB-F108 system, the surface structure of the system changes (Figure 7E): apparently, associates of RB-F108 are fixed on the fine-grained surface of CS in the form of “islands” of irregular shape.

## 3. Materials and Methods

**Figure 8:**
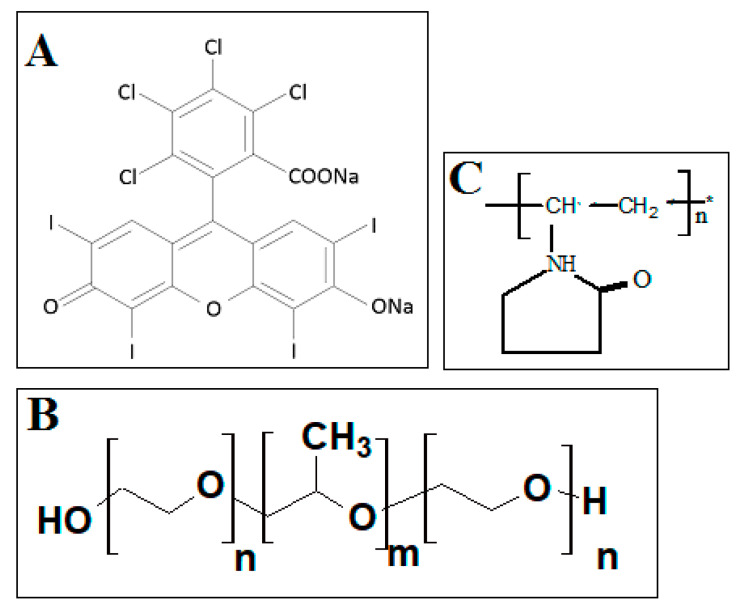
Structural formula of the used reagents: rose bengal (**A**), pluronic F108 (n = 136 and m = 52) (**B**), polyvinylpyrrolidone (**C**).

Rose bengal (RB) (4,5,6,7-tetrachloro-2′,4′,5′,7′-tetraiodofluorescein sodium salt, 95%, Sigma-Aldrich, Burlington, MA, USA) was used as a photosensitizer (Figure 8A). Pluronic F108 (MM 14.6 kDa, Sigma-Aldrich, Burlington, MA, USA) and poly-N-vinylpyrrolidone (PVP) (MM 40 kDa, Sigma-Aldrich, Burlington, MA, USA) were used as amphiphilic polymers (Figure 8B,C). Chitosan (CS) (MM 50–190 kDa, degree of deacetylation of 75–85%, Sigma-Aldrich, Burlington, MA, USA) was used as a polysaccharide; D, L-tryptophan (Trp) (Acros Organics, Geel, Belgium) was used as a substrate.

To prepare the reaction system, rose bengal, AP, and tryptophan were dissolved in phosphate buffered saline (PBS) (pH 7.2–7.6, Sigma-Aldrich, Burlington, MA, USA); chitosan was dissolved in 2% acetic acid. Then the solutions were mixed in certain ratios, and the order of mixing did not affect the activity of the system. The ionic strength of the PBS solutions was *I* = 0.14 mol/L. The tryptophan concentration was 1 × 10^−4^ M, the dye concentration was 2 and 5 × 10^−6^ M, the CS concentration was 0.005 wt.%, the concentration of AP varied from 0 to 1.1 wt.%. The concentration of acetic acid in the reaction mixture containing chitosan was 0.1%. Tryptophan photo-oxidation was carried out in a quartz cuvette (thickness *l* = 1 cm) under stirring and illumination with light with a wavelength of *λ* = 530 nm using an AFS LED phototherapy apparatus (210 mW, Polironic LLC, Moscow, Russia). The kinetics of the process of substrate photo-oxidation was monitored by a decrease in the optical density of absorption bands in the UV spectrum of tryptophan (λ = 280 nm). The effective rate constant ***k****_eff_* of tryptophan photo-oxidation was determined from the linear section of the kinetic curve at the initial time points and was calculated as:

(1) $$k_{eff}=\frac{C_{0}-C_{t}}{C_{0}*\Delta t*C_{RB}}$$

where ***C***_0_ and ***C****_t_* are the initial concentration of the substrate and the concentration of Trp at time ***t***, ***C****_RB_* is the concentration of rose bengal. The number of measurements was at least 10, the measurement error was no more than 10%.

Electronic absorption spectra (EAS) of the dye in the studied solutions were recorded on a Cary 50 spectrophotometer (Agilent, Santa Clara, CA, USA), the RB fluorescence intensity was measured on a Cary Eclipse spectrofluorimeter (Agilent, Santa Clara, CA, USA) (excitation wavelength λ_ex_ = 550 nm).

The degree of fluorescence anisotropy (polarization) (***r***) of pure RB, as well as two component (RB-CS, RB-AP) and three component systems based on it (RB-AP-CS) was automatically calculated by the spectrofluorimeter using the ***G***, ***I****_VV_* and ***I****_VH_* values in the fluorescence emission spectra of the studied solutions according to the formula [49]:

(2) $$r=\frac{I_{VV}-G*I_{VH}}{I_{VV}+2*G*I_{VH}}$$

where ***G*** is the correction factor for the transmission of vertically and horizontally polarized radiation by the monochromator, ***I****_VV_* is the fluorescence intensity for vertical polarization of both excitation and the recorded signal, ***I****_VH_* is the fluorescence intensity for vertical excitation polarization and horizontal polarization of the recorded signal.

To determine ***r***, the studied solutions were excited by light with a wavelength λ_ex_ = 555 nm. The anisotropy of mixtures was recorded at a wavelength λ = 585 nm. The measurement error was no more than 3%.

The pH of the studied solutions (dye–polymer) was determined using a digital HI98103 Checker pH meter (Hanna Instruments, Woonsicket, RI, USA).

NMR measurements were performed using equipment of the Multi-User Analytical Center of the Institute of Problems of Chemical Physics RAS and Science Center in Chernogolovka RAS. The ^1^H NMR spectra were recorded on an AVANCE III superconducting pulsed broadband two-channel NMR spectrometer (Bruker, Billerica, MA, USA) at 25 °C. The operating frequency of the instrument was 500 MHz. The dye, polyvinylpyrrolidone, and their mixture (weight ratio 1:1) were dissolved in deuterated water D_2_O (Sigma-Aldrich, Burlington, MA, USA 99 at. % D) and placed in standard ampoules (outer diameter 5 mm). NMR shift frequencies were calibrated against residual proton signals from deuterated water (4.71 ppm). Registration of NMR spectra was carried out at room temperature 22 ± 0.5 °C.

The structural features of the dye (RB), polymers (F108 and CS), and their component systems (RB-F108 and RB-F108-CS) were studied using atomic force microscopy (AFM) by analyzing the surface of the films. The films were obtained by evaporation on a mica substrate at room temperature in a dust-free cabinet for 16–18 h of aqueous solutions of [RB] = 1 × 10^−3^ M, [F108] = 0.073%, [CS] = 0.1%, (dissolved in 2% acetic acid), RB-F108 (1:10) and RB-F108-CS (1:10:2). For each sample, five films were obtained under the same conditions, and for each film, at least ten measurements were performed on different parts of the surface of the samples. The measurements were carried out in the semi-contact mode (Solver P47, NT-MDT, Moscow, Russia) using cantilevers of the HA-NC ETALON series with a rigidity of 3.5–12.0 N/m and a resonant frequency of 140–235 kHz (curvature radius of 10 nm). Image size was of 10 μm × 10 μm and 3 μm × 3 μm at 1 Hz scan rate with a resolution of 512 × 512 pixels.

## 4. Conclusions

1. It has been shown that effective photosensitizing systems for the generation of singlet oxygen ^1^*O*_2_ based on rose bengal and chitosan (dissolved in 2% acetic acid) are formed when a number of amphiphilic polymers, primarily PVP and pluronic F108, are used as the third component, and buffer medium PBS is used as a solvent. Such RB-AP-CS systems may be promising in the treatment of hard-to-heal purulent wounds and trophic ulcers by aPDT.
2. It was shown that the activity of rose bengal in the generation of singlet oxygen ^1^*O*_2_ in the presence of chitosan decreases due to ionic interaction with protonated CS groups; however, the presence of amphiphilic polymers protects RB from binding to CS, and the photocatalytic activity of the dye is restored. At the same time, if focusing on the rate constant of generation of singlet oxygen ^1^*O*_2_, the efficiency of the ternary system with PVP is higher than the efficiency of the ternary system with pluronic F108.
3. The introduction of chitosan into an aqueous and PBS solution containing RB led to a decrease in the optical density of both absorption bands in the EAS and the RB luminescence intensity, which is associated with the RB-CS ionic interaction and the formation of the lactone photoinactive form of RB.
4. The formation of RB-AP complexes was confirmed by the methods of electron and fluorescence spectroscopy. In particular, when pluronic F108 was introduced into a solution containing RB and CS, a bathochromic shift of the absorption band by 20 nm and the fluorescence band by 15 nm (λ = 550 nm) in the EAS of RB was observed.
5. The effect of polymers on the degree of fluorescence anisotropy (***r***) of RB was studied. It was shown that in the presence of chitosan, an increase in ***r*** is observed, which is obviously associated with the formation of ionic bonds between RB and chitosan.
6. The addition of pluronic to the RB-CS system in aqueous and PBS media leads to a decrease of the degree of RB fluorescence anisotropy (***r***), which may be due to the dispersal of RB molecules in pluronic F108 micelles. In the presence of PVP, which does not form micellar structures in water, the effect of reducing the value of ***r*** was not observed.
7. The presence of intermolecular interactions of RB with PVP was shown by the proton NMR method. In addition, the formation of RB-F108 macromolecular complexes, which form associates during solution concentration (in particular, during evaporation), was shown by AFM.

## Figures and Tables

**Table 1:** Effect of chitosan and acetic acid on the absorption spectra of RB in PBS and water.

	Absorption			
	RB in PBS		RB in Water	
**RB**	**D_max (λ = 550 nm)_**	**D_shoulder (λ = 515 nm)_**	**D_max (λ = 550 nm)_**	**D_shoulder (λ = 515 nm)_**
	0.50	0.16	0.50	0.16
**CS (0.005%)**	0.25	0.11	0.24 ^(^**^)^	0.15 ^(^**^)^
**AcOH (0.1%)**	0.32	0.12	0.03 ^(^*^)^	0.01 ^(^*^)^
**AcOH (0.25%)**	0.09	0.04	-	-
**AcOH (0.35%)**	0.05	0.03	-	-

[RB] = 5 × 10^−6^ M. (*)—D**_max_** (λ = 565 nm) и D**_shoulder_** (λ = 535 nm). (**)—D**_max_** (λ = 555 nm) и D**_shoulder_** (λ = 520 nm).

**Table 2:** Effect of chitosan and acetic acid on the fluorescence intensity of RB in PBS and water.

	**Fluorescence**			
	**RB in PBS**		**RB in Water**	
**RB**	**_λmax_**	**Intensity**	**_λmax_**	**Intensity**
	568	252	568	241
**CS (0.005%)**	568	98	568	60.5
**AcOH (0.1%)**	569	180		9.5
**AcOH (0.25%)**	566	79.5	-	-
**AcOH (0.35%)**	566	39	-	-

**Table 3:** Fluorescence anisotropy (r) of RB in the absence and presence of AP and chitosan. The excitation wavelength is 555 nm.

Sample	*r* of the Samples in Aqueous Solution (585 nm)	*r* of the Samples in PBS Solution (585 nm)
**RB**	0.26 ± 0.01	0.27 ± 0.01
**RB-AcOH**	0.26 ± 0.01	0.26 ± 0.01
**RB-CS**	0.32 ± 0.01	0.28 ± 0.01
**RB-F108**	0.27 ± 0.01	0.27 ± 0.01
**RB-F108-CS**	0.09 ± 0.01	0.23 ± 0.01
**RB-PVP**	0.31 ± 0.01	0.31 ± 0.01
**RB-PVP-CS**	0.23 ± 0.01	0.31 ± 0.01

[RB] = 5 × 10^−6^ M, [AcOH] = 0.1%, [CS] = 0.005%, [F108] = 0.15%, [PVP] = 0.2%.

## Data Availability

The data presented in this study are available on request from the corresponding author.

## Sample Availability

Samples of the compounds are available from the authors.
